# Adaptation of Soil Physical Measurement Techniques for the Delineation of Mud and Lakebed Sediments at Neusiedler See

**DOI:** 10.3390/s131217067

**Published:** 2013-12-12

**Authors:** Ilse Kogelbauer, Erwin Heine, Christopher D'Amboise, Christoph Müllebner, Wolfgang Sokol, Willibald Loiskandl

**Affiliations:** 1 Institute of Hydraulics and Rural Water Management, University of Natural Resources and Life Sciences, Vienna, Nuβdorfer Lände 11, Vienna 1190, Austria; E-Mails: christopher.damboise@gmail.com (C.D.); christoph.muellebner@gmx.at (C.M.); wolfgang.sokol@boku.ac.at (W.S.); willibald.loiskandl@boku.ac.at (W.L.); 2 Institute of Surveying, Remote Sensing and Land Information, University of Natural Resources and Life Sciences, Vienna, Peter-Jordan-Straβe 82, Vienna 1190, Austria; E-Mail: erwin.heine@boku.ac.at

**Keywords:** sediment profiles, mud layer delineation, shallow steppe lake, cone penetrometer, FDR, echo sounding

## Abstract

For many water management issues of shallow lakes with non-consolidated sediments hydrographic surveys of the open water area and reed belt areas are required. In the frame of water management strategy for the steppe lake Neusiedler See, located between Austria and Hungary, a hydrographic survey was conducted. In the open water area (water depth ≥1 m) a sediment echosounder was used. To validate these measurements and to distinguish between water, mud, and sediment layers in the shallow lake and reed belt area additional measurements were needed. As no common standard methods are available yet, we developed a measurement system based on two commonly applied soil physical measurement techniques providing reproducible physical values: a capacitive sensor and a cone penetrometer combined with GNSS-positioning enable dynamic measurements of georeferenced vertical water-mud-bedsediments profiles. The system bases on site-specific calibrated sensors and allows instantaneous, *in situ* measurements. The measurements manifest a sharp water-mud interface by a sudden decline to smaller water content which is a function of the dielectric permittivity. A second decline indicates the transition to compacted mud. That is concurrently the density where the penetrometer starts registering significant penetration resistance. The penetrometer detects shallow lakebed-sediment layers. Within the lake survey this measurement system was successfully tested.

## 1. Introduction

There is a common interest on mapping and studying the bed constitution of natural water bodies, artificial harbours, or inland waterways for water management issues or navigability of shipping pathways, especially at the presence of a mud layer rich in fine-grained sediments. In the past, this non-consolidated, near-bottom mud layer was only assigned to few locations in channels, harbours and bays, but it is also a ubiquitous phenomenon in any natural water body [1]. It is present in any natural water body with sufficient supply of fine-grained sediment and periods of low flow velocity such as lakes and estuaries.

Acoustic techniques are extensively used in hydrographic surveys for lakebed mapping as they provide relatively rapid coverage of large lakebed areas compared to direct sampling methods [2,3]. But the inherent problem at the presence of a mud layer is the acoustic delineation and mapping of the lakebed surface. The mud density is slightly higher than that of water and increases gradually with depth [4], hence the impedance contrast offered to an acoustic wave by the water-mud-lakebed interface is less significant than by a water-lakebed interface. To overcome these difficulties of lakebed mapping McAnally *et al.* [4] emphasized the research need for improving or combining existing measurement techniques.

To support acoustic techniques for mud layer and lakebed mapping complementary methodologies with a soil physical approach are recommendable and have already been applied [1,3,4]. However, these methods require intensive sampling effort. So far there is no common standardized method that delineates water, mud and consolidated lakebed sediments at the presence of a distinctive transition zone from water to lakebed. Many studies reported the development of sensors that combine cone penetrometer with water content measurement systems such as time domain reflectrometry (TDR) or time domain transmissometry (TRT) [5–7]. All these presented probes and methods were developed for agricultural or mountainous forested soils [6,7], but not for surveying the challenging environment of a lake. Therefore some inadequacies of these probes for the intended application were their standardization, lack of ruggedness, accuracy of penetration resistance *PR* measurement and obviously the very small maximal measurable depth of 40 to 60 cm.

Thus, the purpose of the study was the adaptation of commonly used and well-known soil physical measurement techniques for the *in situ* delineation of mud and shallow lakebed-sediment layers within a hydrographic survey. This was accomplished by: (1) establishing a methodology; (2) the laboratory calibration of the sensors; (3) a measurement campaign with concurrent refinement of equipment, software, and measurement systematic; and (4) analyzing raw data to delineate mud and lakebed-sediment layers.

The proposed measurement system consisted of a capacitive sensor determining the water content in soft mud and a cone penetrometer measuring the penetration resistance *PR* in compact mud layers and lakebed sediments. It was joined together with a Global Navigation Satellite System (GNSS) Real-time Kinematic (RTK) positioning for dynamic, vertical point-measurements precise in location. The system of combined techniques enabled instantaneous, *in situ* survey to provide georeferenced, vertical profiles for mud and lakebed delineation of shallow water bodies with consolidated bed sediments. With this system many points high in their information quality could rapidly cover large lakebed areas with sufficient spatial resolution, but without extensive sampling effort.

This measurement system was applied within a hydrographic survey of the shallow steppe lake Neusiedler See and its surrounding reed belt located in the Pannonian Basin, along the border between Austria and Hungary. It was used as complementary measurements to validate echo sounding data, and to survey the very shallow water zones of the open water area (water depth ≤1 m) as well as the surrounding reed belt. The hydrographic survey aimed to provide data for the water and reed management of the lake. Generally, the water management of the Neusiedler See is rather challenging in a focus on its extraordinary uniqueness together with multiple utilization interests such as water sports, tourism and agriculture.

## 2. Methodology

### 2.1. Design of the Measurement System

**Figure 1.**
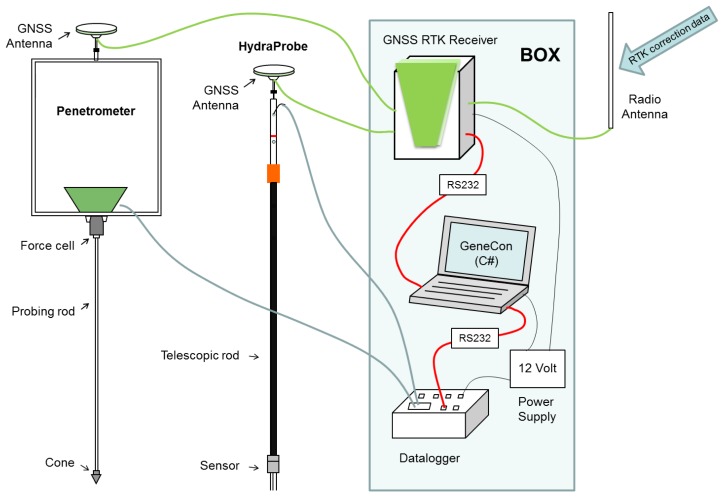
Scheme of the measurement system: sensors and electronic equipment for data acquisition (GNSS RTK receiver, data logger, notebook running GeneCon-software and power supply) stored in a splash-proof water-tight box.

The main aim of the designed system is the delineation of water, mud, and lakebed-sediment layers at the Neusiedler See. The system utilizes three main components (Figure 1):

- Sensor System: it consists of two well-known soil physical measurement techniques, a capacitive sensor (Hydra Probe, Stevens Water Monitoring System, Portland, OR, USA) and a modified penetrometer (Eijkelkamp, Giesbeek, Netherlands) [8] that measures water content and soil penetration resistance *PR*.
- Data acquisition system: a data logger (CRX23, Campbell, North Logan, UT, USA) is used to collect and process data from the sensors and a GNSS RTK (GNSS RTK receiver/System 1200/Viva/GS25, Leica, Heerbrugg, Switzerland).
- Software: the software is created to synchronize sensor data with GNSS position and convert it to a desired file format for further application.

The sensors are used consecutively at the same site to create instantaneously a vertical profile in the soft mud and the consolidated lakebed sediments. Each sensor is synchronized with position information from the GNSS System 1200/Viva/GS25 RTK System from Leica with accuracy of about ±2 cm [9] using our therefore developed software tool GeneCon. Our method's advantages are obliviously precise location (x,y,z) ±3–5 cm in overall accuracy, dynamic *in situ* point-measurements and comparability due to values based on physical units.

#### 2.1.1. Hydra Probe

The Hydra Probe (Stevens Water Monitoring System), is a dielectric permittivity sensor based on frequency domain reflectometry (FDR) at 50 MHz and indirectly indicates volumetric water content θ (m^3^·m^−3^) (sensor description see [10]). Usually it is applied for soil water monitoring in unsaturated conditions such as agricultural areas. For our application it is fixed on a telescope rod with a GNSS antenna mounted on top and used in saturated conditions to delineate air, water, and soft mud.

The Hydra Probe with analog output is used, because its rate of several measurements per second is faster compared to its digital counter parts (SDI-12 and RS-485) [11]. The output is transformed into the parameters (soil temperature measured by an internal thermistor, soil electrical conductivity, and dielectric permittivity). The directly measured relative complex dielectric permittivity, which composes of a real and an imaginary part, is further temperature corrected [12] and correlated to the soil water content [10]. The temperature corrected real part of the relative complex dielectric permittivity ε*_rcorr_* enables the layer delineation by its significant difference; with *ε_r_* in air (1), Neusiedler See water (70–80), and solid particles (4–7).

It is proven that the relationship of probe length submersed in suspension and its output *ε_rcorr_* is highly linear [13] and thus an advantage. The sensor's reference point is set at the pins protruding base.

#### 2.1.2. Penetrometer

Cone penetrometers are recommended in the American Society of Agricultural Engineers (ASAE) Standards as a standard method for measuring the penetrometer resistance *PR* of soils [14]. They are extensively used to evaluate soil strength especially in agriculture for resistance to root penetration [15], soil trafficability and soil compaction [16,17], but also for hydrogeomorphological surveying to identify depth of boundaries between layers [7]. In our case it should indicate dense layers to detect the lakebed surface and the shallow sub-bottom strata in combination with the sediment echosounder.

A penetrometer (Eijkelkamp, Art. Nr. 06.15.01) is modified twofold: the strain gauge is directly connected to the datalogger (Campbell CRX23) and the length of the probing rod is extended. A GNSS antenna is mounted on top of an aluminium frame, which is fixed on the splash-proven box accommodating the electrical components. The GNSS antenna is exactly aligned with the cone tip. The cone tip has a top angle of 60°, a diameter of 20.60 mm with a cross-sectional cone area *Ap* of 3.33 cm^2^ and a cylindrical shaft reduced in diameter to 20.08 mm [8]. The probing rod extensions are not exceeding the shaft with 14 mm in diameter and can be assembled by individual items of 0.5 and 1 m up to 5 m length.

The strain gauge, the central component of the penetrometer, is a force transducer returning output voltage, which differs from the basic voltage when deformation (either tension or compression) induces resistance changes. The output voltage is related to the weight force by laboratory calibration and measures the penetration resistance. The probing rod is fixed on the force transducer and can be put together for variable length, depending on the sludge depth. The maximum penetration resistance capacity of the force transducer is limited with 1,000 N.

The pressure resistance *PR_x_* related to a penetrometer cone of a specific size is described by Equation (1) stated by Bradford [18]:

(1) $$PR_{x}=F_{p,x}/A_{p,x}$$

where *F_p,x_* is the force required to push the penetrometer through the soil and *A_p,x_* is the cone's projected cross-sectional base area in cm^2^.

The *PR* is influenced not only by the soil physical properties but also by the probe characteristics governing the friction. Friction inducing factors are the cone angle, the diameter of cone and shaft, roughness and penetration rate [18]. The *PR* varies with soil factors such as bulk density, matric potential or water content, texture and organic matter, but is mainly influenced by the water content and bulk density [19]. Kosugi *et al.* [7] stated that the dependence of the *PR* on the soil water content may mask the correlation of *PR* to other soil properties; however this dependence is less prominent for the inherent saturated conditions at the measurement site.

#### 2.1.3. Data Acquisition System (DAS)

**Figure 2.**
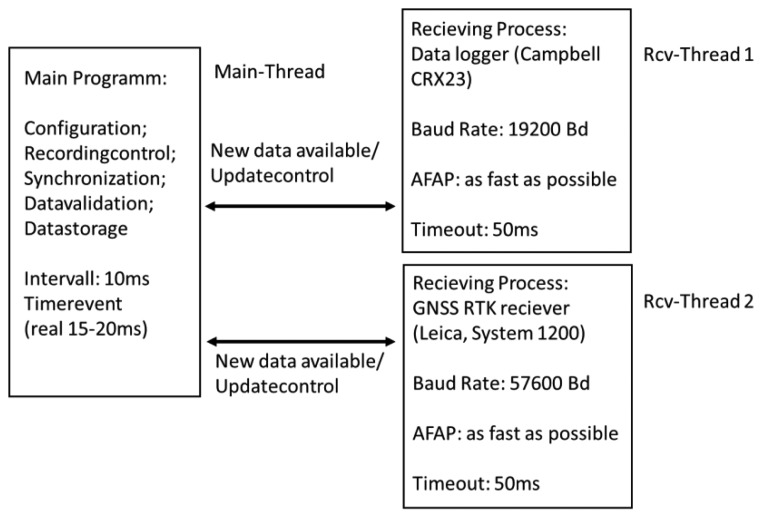
Flow chart of data processing.

Both sensors, the Hydra Probe and the Penetrometer, are connected to a Campbell CR23X data logger (sampling interval of 0.1 s; data transfer RS232). Data from the data logger and the GNSS RTK receiver (NMEA data specification, 0.1 s data rate) are synchronized and stored with a C# based software tool running in an attached notebook (Figure 2). The software is the core of the DAS, providing communication, control and validation functions. Software specifications are: command line; C# with .NET4.0 or .Net3.5 Framework, WinXP or Win7 as operating system, Visual C# 2008 Express Edition as development tool. The software contains three threads. One thread is responsible for receiving data from the Campbell CR23X logger (Rcv-Thread 1). The second thread receives data from the GPS 1200 (Rcv-Thread 2). The third and main thread is primarily responsible for synchronizing and storing the received data of equal time (Main-Thread). To make sure that only the newest data are processed the input buffer of the receiving threads are cleared after fetching data and the time out for receiving new data is set to 50 ms. The baud rate for the two RS232 ports are set to 19,200 Bd (CR23X) and 57,600 Bd (GPS 1200). During the C# software tool development it is ascertain that the latency time of the GNSS RTK System for providing the latest position data stays below 200 ms. An acoustic signal announces that the data logger is ready and that the measurement can start. The signal indicates by its pitch the GNSS position quality and by its beat the data receiving interval. The power source and the electronic equipment are stored in a splash-proof box (see Figure 1).

GNSS accuracy is increased by using RTK-correction signals which are emitted via radio antenna from GNSS base stations either in the North West or the South East of the lake.

### 2.2. Study Site

The Neusiedler See is the westernmost steppe lake in Central Europe surrounded by an extensive reed belt. It is located on Austrian and Hungarian territory in the flat terrain of the western part of the Little Hungarian Plain. The lake basin covers a total area of 321 km^2^—at reference water level elevation 116.50 msl (above Adriatic mean Sea level) [20]—including a reed belt of about 178 km^2^ overgrown with *Phragmites australis* [21]. The lake is very shallow, with water depths less than 2 m. The lakes predominant characteristic is a huge stratified mud layer of mainly fine-grained sediments, which is a distinct transition zone between water and the lakebed.

The water balance of the lake is dominated by precipitation and evaporation, and a natural inflow is either lacking or minor. Due to these facts the lake is prone to strong water level fluctuations and even to draught conditions, predominantly in summer at high evaporation rates [22]; however human interference, especially by water level regulation, is aside from climate the main source of desiccation and ecological consequences. Since the water level regulation in 1965 at the artificial weir within “Einserkanal”, the only outflow (artificial) of the lake, an almost constant water level is reached. Within the period 1963–1988 almost doubling in mud volume on Austrian territory (75 Mio·m^3^ to 150.17 Mio·m^3^) was shown by Bácsatyai *et al.* [20]. The increase in sediments on Austrian territory is enhanced by wind-driven sediment shift and accumulation in the reed belt; silting up is even more intensified by massive increase of the reed belt area during 1901 to 1963 [20].

### 2.3. Sensor Calibration

#### 2.3.1. Hydra Probe Calibration

The Hydra Probe calibration is particularly recommended for very wet soil with water content >60% [23]. Hence it was calibrated for wet mud from the Neusiedler See relating the temperature corrected real part of the relative dielectric permittivity *ε_rcorr_* and the gravimetric measured volumetric water content *θ* (m^3^·m^−3^) [24]. To account for the variation of mud composition throughout the lake, four mud types were considered in the calibration procedure. They were different in their particle size distribution (from clay to loamy sand) and organic matter with varying degree of decomposition. Two calibration methods were performed to gain a large range of water contents from compact to soft mud.

The calibration set of the first method M1 consisted of three mud samples taken from the South East edge of the lake: the first sample was taken from an organic rich top layer with many reed roots and plants, which was water saturated while sampling but subjected to periods of drying out during the year. Its particle size distribution is 21.66% sand (0.05 mm to 2 mm), 25.37% silt (0.002 mm to 0.05 mm) and 52.97% clay (below 0.002 mm). The second one was sampled from the more sandy layer with higher decomposed roots that lies just below the first one. The third sample was taken from about 1.5 m below the water surface from the lake bottom in an area close to an artificial canal; it contains 78.28% sand, 7.94% silt and 13.78% clay (average of five replicates). The second and third sample are similar in terms of texture and firmness.

Plastic jars of approximately one liter size were filled two-thirds full; to some jars dry mud or lake water was added and well mixed for more than 15 h with an electric tumble mixer to ensure homogeneity. The sensor was inserted into the samples ensuring that all the pins were completely covered. After the measurement the mud was filled in cylindrical metal rings of 200 cm^3^ (diameter 7 cm, height 5 cm) and oven dried at 105 °C until mass constancy to measure the gravimetric water content and further calculate the volumetric water content *θ*.

The second method M2 of calibration used mud samples extracted from deeper layers via a core sampler, where vacuum suction keeps the sample in the plastic tube. It was taken at the Eastern Side close to the reed belt at about 0.5 m water depth. The mud contains 26.18% sand, 32.75% silt and 41.07% clay. A 2 l-beaker was filled with mud until the mark of 600 mL and topped up with lake water until 700 mL and mixed with a spoon. Then the sensor was completely submerged in the mixture for the measurement. Again, the oven dried mass (at 105 °C and mass constant) was determined to calculate *θ*. For broader range of water content the process was repeated 5 times using about 100 mL less mud (and 100 mL more lake water) for each run.

A third degree polynomial (Equation (2)) was fitted to the data pairs of *ε_rcorr_* with *θ* obtained by both methods M1 and M2 (*R^2^* = 0.9877) [24]. To fix the whole water content spectrum the lake water and oven dried ground mud were used as fixed points with assumed volumetric water content of 1 m^3^·m^−3^ and 0 m^3^·m^−3^ respectively. The Hydra Probe measurement error of *ε_rcorr_* was ±1.5% or ±0.2 m^3^·m^−3^ whichever was greater. The measurement error of *θ* depend on the particle size, it is ±0.01 m^3^·m^−3^ or ±0.03 m^3^·m^−3^ for fine textured soils using the calibration provided by Stevens Water Monitoring System, Inc. [23]. The resulting calibration error for our application was ±0.2 m^3^·m^−3^:

(2) $$\theta =-0.087968+0.027307\;\varepsilon_{r}-0.000356\;{\varepsilon_{r}}^{2}+2.34\times 10^{-6}{\varepsilon_{r}}^{3}$$

**Figure 3.**
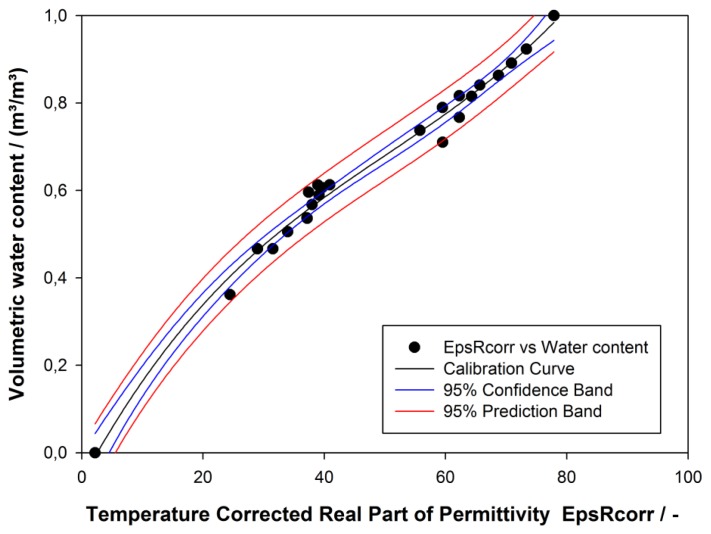
Fit of calibration function with a 3rd degree polynomial (all data points) [modified 24].

The clustering of similar points in Figure 3 obviously demonstrated that the mud composition had a significant influence on *ε_r_–θ* relation. The mud composition varies throughout the lake in terms of organic matter content and its degree of composition, the particle size distribution, water saturation in periodically flooded areas. However, it was also recognized that highly organic mud samples had lower liquid consistency compared to its high water content than those samples with low organic content. The reason is probably due to a large fraction of water not free in soil pores, but retained in structures such as plant cells [24].

Investigations on the relation of water content and organic matter content need to be done to improve the exact determination of the water content. In our application the relative change of water content was sufficient for the layer delineation—water, mud, and bed sediment layers—as long as there was a significant change in *ε_r_*.

During field application at the beginning of the campaign, it was noticed that dried mud covering the pins reduced *ε_r_* readings significantly from about 0.75 to 0.6 for lake water. Hence, the sensors need to be cleaned carefully before the first measurement.

#### 2.3.2. Penetrometer Calibration

The laboratory calibration relates the output voltage of the force transducer induced by the applied force. Therefore a removable wooden top part 20 × 20 cm in dimension was fixed in the opening where the probing rod was connected with the force transducer. The maximum load-bearing capacity was 45 kg. The force transducer was loaded with weight, left there for several seconds to eliminate randomly fluctuations. The base signal included the weight of the wooden top part of 535.3 g. A weight (5082 + 535.3 g) was arranged centric and eccentric (nine positions in total). The mean average output voltage of 205.7 mV for eccentric loading only varied ±0.2 mV from centric loading. The mean standard deviation was 2.7 mV which is 1.3% of total voltage and also addresses the error for eccentric loading, which may occur under field conditions [25].

To obtain the linear calibration function (Equation (3)) the load of the force transducer was stepwise increased to 45 kg and afterwards reversely decreased:

(3) $$y=29.82x+y_{0}$$

where *y* (mV) is the output voltage for the applied mass *x* (kg) with a changing offset voltage *y_0_* (mV) and 29.82 ± 0.2 mV·kg^−1^ as slope.

The influence of the probing rods weight was crosschecked. The output voltage induced by the tension force of the hanging rod of the penetrometer was compared with the voltage calculated by the calibration function. It was shown that also the tension force (negative voltage) was in a linear relation with 29.82 ± 0.2 mV·kg^−1^ to the output voltage [25]. The offset voltage of 18.40 mV marginally shifted since its first operation, which might stem from a hysteresis due to material fatigue from the strain gauge. Therefore the base signal was identified every time starting an operation.

Further, the penetration resistance *PR* in MPa was established based on Equation (1) using the calibration function (Equation (3)) with an additional factor *R_p,x_* (mV) for the weight of the probing rod:

(4) $$PR_{x}=\left(\frac{y-y_{0}+R_{p,x}}{29.82\times A_{p,x}}\right)\times 0.09807$$

We applied a conversion proposed by Bradford [18] to obtain the *PR_x_* in MPa from the output voltage. Hence, the factor 0.09807 implies the conversion from kg·cm^−2^ to MPa including gravity. The factor *R_p,x_* for the additional probing rod weight was considered to be 63.71 ± 21.24 mV for an average length of 3 ± 1 m. The marginal error evolving from that varying rod length of ±0.2 MPa or 2.1% was therefore tolerated.

### 2.4. Field Data Collection

The designed system was applied from a small boat for investigations at the open water surface and at the reed shore line whereas the reed belt was investigated with an adopted reed cutting machine. At the open water three replicates were made with both sensors to cover spatial variability. But to cover the lake-reed transition at least two measurements with each sensor where taken in the reed, at the edge and the water within a range of 6 m (boat length). In the reed at least three replicates with each sensor were measured at one spot. Mud core samples for soil physical and geological analyses and for the verification of the sensors' measurements were taken at specific spots.

## 3. Results and Discussion

### 3.1. Hydra Probe

The temperature corrected real part of the relative dielectric permittivity *ε_rcorr_* (*EpsR_corr_*) measured by the Hydra Probe (HP) supports the layer delineation in a murky suspension due to the significant difference in *ε_r_* of air, water, and solid particles. In our case of saturated conditions the measured volume only consists of solid particles and water. Thus *ε_r_* plainly indicates the fraction of solids and water due to the significant difference in *ε_r_*, which is 3 for dry soil and about 80 for lake water. Variations of *ε_rcorr_* in the lake are induced by the temperature, but even more by the inherently high electrical conductivity *EC*. The *EC* is higher than 2,000 μS/cm with maxima at about 3,000 μS/cm and induces *ε_rcorr_* to vary from 65 to 80.

**Figure 4.**
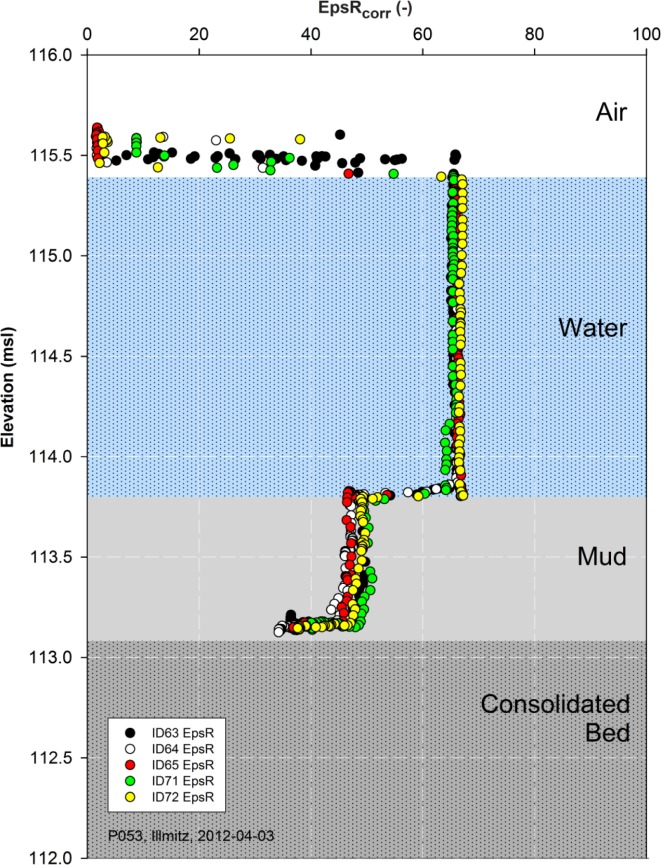
Hydra Probe measurements are used for layer indication of air, water, mud, and bottom bed. A steep decline in *ε_rcorr_* (EpsR_corr_) indicates the water-mud interface. Another decline occurs when mechanical resistance of compact mud and the lakebed sediments prevent further submersion. Point P053.

In Figure 4 unfiltered data of five replicate Hydra Probe measurements at point P053 in the open water area (Illmitz; Universal Transverse Mercator (UTM) coordinate system: 33N 629812 5291320) explains the inherent measurement procedure. Each measurement starts in air, so the current water surface can be observed when the sensor is submerged into water, which is a valuable side effect. Further submersion in water at almost constant *ε_rcorr_* of 66 (occurring range 65 to 80) is interrupted by a sudden decline in *ε_rcorr_* to about 48 (occurring range 45 to 60) indicating the water-mud interface (mud surface). Then the ongoing submersion continuous in the mud layer till the mechanical resistance prevents further penetration of the sensor in more compact mud layers and the lakebed sediments.

At the water-mud interface the sensor pins are fully submerged in the mud and the base of the sensor's cylindrical body is aligned with the interface. Consequently, it is at the decline's low *ε_rcorr_* value. The *ε_rcorr_* in the mud layer is almost linear, just slight variance due to spatial variability occurs.

The same trend is given for all replicates (Figure 4). The air-water interface (water surface) is at 115.39 msl (above Adriatic mean Sea level), the water-mud interface (mud surface) is at 113.80 msl and the deepest point of submersion is at 113.16 msl, all as average of five replicates. Thus the mean thickness of water and soft mud are 1.59 m (±0.01 m) and 0.64 m (±0.03 m), respectively. In air the measurements are diverging slightly, a phenomenon related to the moving barge with the waves and is in an average range of 0.16 m (±0.04 m) (Table 1).

**Figure 5.**
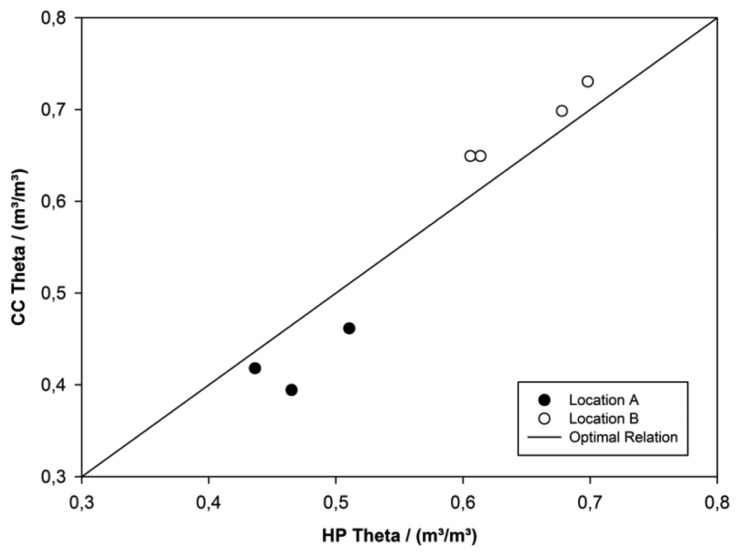
The field validation compares the volumetric water content determined with the calibrated Hydra Probe (HP Theta) and gravimetrically determined from cylindrical cores (CC Theta). HP Theta is related with the directly measured EpsR_corr_ by our calibration function. Two locations different in core size distribution and root content at the shore of the reed belt were sampled (A, B).

The Hydra Probe calibration function determined in the laboratory was validated in the field at two random locations at the reed belt shore adjacent to the lake. At each location core cylinder samples were taken next to the *in situ* measurements with the Hydra Probe (three replicates). Location A was sandy with lots of fine reed roots (mm in diameter) compared to Location B high in silt and clay with many medium roots (diameter approx. 0.5–1 cm) causing numerous holes containing water. The agreement between the volumetric water content determined by the Hydra Probe (HP Theta) and gravimetrically determined from cylindrical cores (CC Theta) was checked (Figure 5). Due to spatial heterogeneity small deviations were evident, but still staid in a reasonable average limit of −0.047 in Location A and 0.026 in Location B. In A HP Theta overestimated CC Theta while in B it underestimated. This phenomenon can be explained due to non-optimal conditions of core sampling (Figure 5). But also volume reduction by shrinking, weight reduction due to lower particle density of organic material, and roots containing water were observed.

### 3.2. Penetrometer

Pressure resistance *PR* is the significant factor for detecting lakebed surface and shallow lakebed-sediment layers. A sharp increase in the *PR* of the penetrometer indicates a compact layer, much more compact than a soft mud layer where the *PR* is too low to be detected.

**Figure 6.**
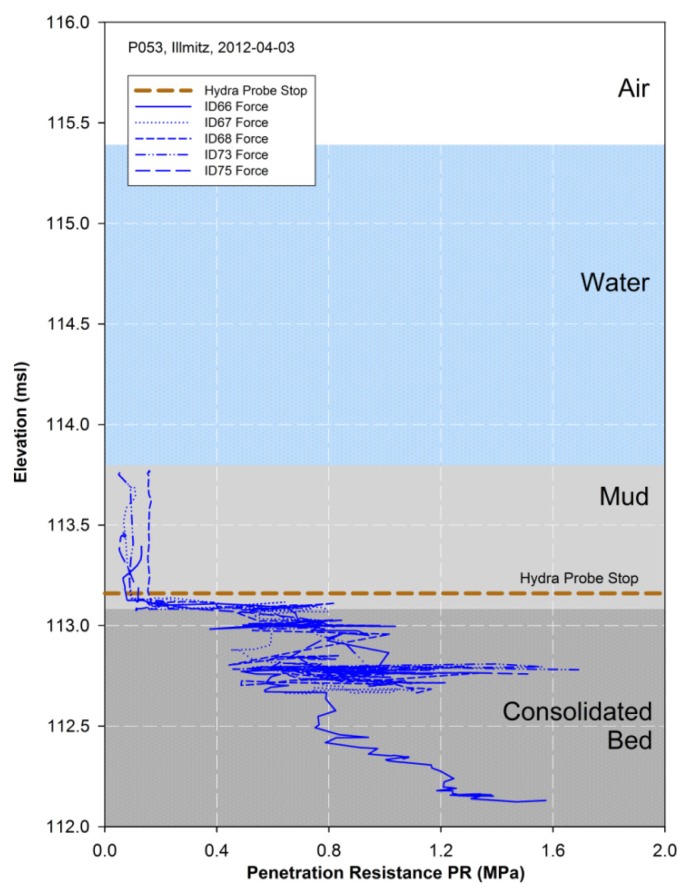
Penetrometer measurements (five replicates) are used for the detection of lake bottom and shallow sub-bottom strata in Point P053. A significant relative change in *PR* indicates layers different in their density or degree of compaction. The detection of significant *PR* coincides with the submersion end of the Hydra Probe.

In Figure 6 raw data of five replicate measurements at the same point P053 as for the Hydra Probe measurements are shown. Each penetrometer measurement starts in water or soft mud at minimal pressure resistance and rapidly increases when reaching more compact shallow layers with higher density. The point of detecting significant *PR* coincides with the submersion end of the Hydra Probe sensor, thus a continuous vertical mud-sediment profiling is gained with both sensors (Figure 6). Layers of high or low density can occur in between the shallow top layer and the very end of penetration. At point P053 the mud-lakebed interface (lakebed surface) is at 113.08 msl (±0.01 m), which results in a final mud layer thickness of 0.72 m (±0.02 m) (Table 2). Another consolidated lakebed-sediment layer is at 112.78 msl (±0.02 m). Nevertheless, the detection of shallow layers is achieved by this setup although maximal rod length of up to 5 m and rod diameter are restricted to manual operation. Besides the probing rod length, the lateral bending resistance of the rods, the highest possible applied pressure, and man power are other external factors limiting the measurement depth.

The penetrometer graph (Figure 6) comprises only raw data visibly quite unsteady, that arises from handling, and needs some additional filtering. Inconsistency in elevation is mainly observed at dense layers when breakthrough and rapid falling is accompanied by interruption of GNSS data processing. The reason is the acceleration in submersion-velocity, because the GNSS RTK receiver generates precise elevation data of dynamic processes by forward calculation assuming constant velocity. It appears as loops that can easily be differentiated from soft layers for two reasons: a very small elevation difference of few cm and the lack of GNSS elevation data when the pressure readings jump back. Moreover short term relief evolves when the person changes position while pushing the penetrometer down. However, dense layers are mainly identified by a significant relative change in *PR* and less due to defining absolute values of *PR*.

### 3.3. Validation of Echo Sounding in the Open Water Area

For the acoustic mapping of the lakebed at the Neusiedler See a Single Beam Echosounder (SBES) at frequencies of 710 kHz, 200 kHz, and 100 kHz and additionally a Sub Bottom Profiler (SBP) at 10 kHz were used at the open water area [26]. The applied SBES, a parametric acoustics unit of the Innomar SES-2000 sediment echosounder, gave an excellent resolution and good penetration into the lakebed at water depth ≥1 m [27]. The result is a continuous, real-time displayed record of bathymetry that illustrates the first significant reflecting interface in the water-mud transition and further reflecting sediment interfaces between sub-bottom strata [2]. The reflection of acoustic energy takes place at interfaces of differing acoustic impedance, and the reflection strength depends on the degree of impedance contrast [2]. The inherent problem at the presence of a mud layer is the acoustic delineation and mapping of the mud and lakebed surface due to low impedance contrast explained by small density gradients. Thus the complementary, soil physical measurements support the layer delineation of the echo sounding.

SBES and SBP tracks were measured in a raster from (500 m × 100 m) in the open water areas at a minimum water depth of 30 cm (SBES) and 1 m (SBP). About 60 validation-points were selected for the soil physical measuring campaign in spring 2012 based on the echosounder tracks. At each validation-point at least 2 to 3 replicate measurements with the Hydra Probe and the penetrometer were taken to detect possible spatial variability.

**Figure 7.**
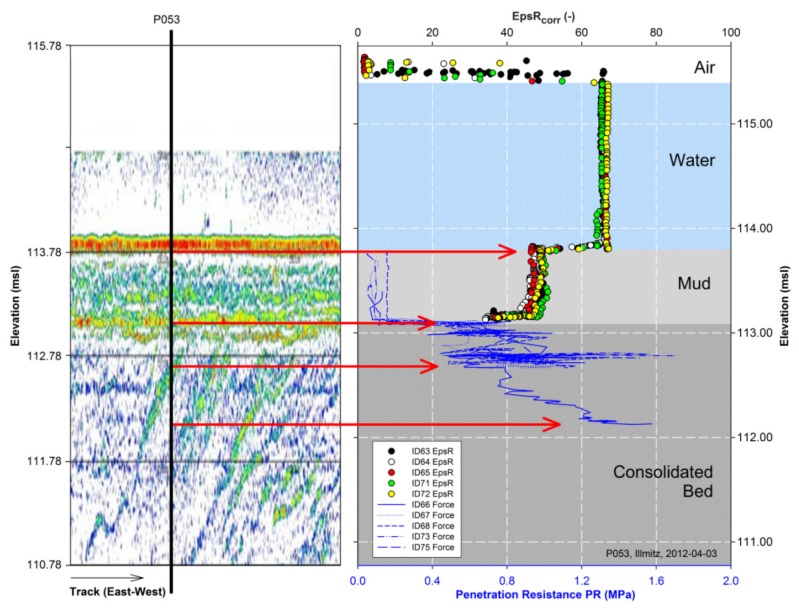
Example of preliminary layer validation by correlating the SBP echogram sequence (**Left**) including the validation-point P053 with the soil physical measurement system (**Right**). The layering in the echogram indicated by red-yellow, which visualizes strong reflections due to high impedance contrast, coincides reasonable well with the soil physically determined interfaces.

The low frequency SBP echogram sequence (Figure 7) shows an example of a layer validation. The echogram color pattern visualizes differences in the density of sediment layers by a colour scale from blue (weak reflections) to red (strong reflections). The vertical line in the echogram shows the location of the validation-point P053 of the soil physical measurements. The blank top part of the echogram (115.78–114.78 msl) evolves due to the minimal operation depth of the SBP in the water body. The first significant reflection follows at height 113.93–113.78 msl in red-yellow colour that coincides with the mud surface detected at 113.80 msl by the Hydra Probe. Further significant reflection signal coincides with the lakebed surface at 113.08 msl. Beneath that, a significant reflection coincides with an increase in *PR* at 112.78 msl. Overall in point P053 a good correlation of the echogram with the soil physical measurement data—Hydra Probe and the penetrometer—is demonstrated.

## 4. Conclusions

A new measurement system for delineating mud and lakebed sediments was developed and successfully tested. The new set up enables instantaneous measurements of water content and penetration resistance at shallow water bodies, which are rich in non-consolidated fine-grained sediment layers. Georeferenced vertical profiles for layer delineation of water, mud, and shallow lakebed sediments are obtained. The system provides a high information quality at a measurement point and benefits from the combination of two already standardized soil physical methods that support comparability and reproducibility. Moreover, it is less time consuming and requires minimal sampling effort when covering large areas in sufficient spatial resolution. However, in data interpretation and interface delineation significant relative changes in the physical values—*ε_rcorr_* and *PR*—are used rather than absolute values. It was shown that the validation of echo sounding data was supported by this adapted measurement system for the application at the steppe lake Neusiedler See. Generally, it worked out well and the good correlation proposed further data validation. Finally, the developed methodology offers an adequate and reliable *in situ* approach for surveying campaigns to delineate and map mud and lakebed sediments.

## Figures and Tables

**Table 1.** Mean interface elevation (msl) and mean layer thickness (m) indicated by changes in *ε_rcorr_* that delineate air, water, and mud in point P053.

	**Interface**			**Layer Thickness**	
	
	**Elevation (msl)**	***ε****_rcorr_*** (−)**		**Thickness (m)**	**Δ *ε****_rcorr_* **(−)**
Air-Water a	**115.39** (0.01)	65.75 (0.61)	Waves	**0.16** (0.04)	
Water-Mud	**113.80** (0.01)	48.77 (0.92)	Water a	**1.59** (0.01)	16.98 (0.90)
HP Stop b	**113.16** (0.03)	37.03 (1.88)	HP Mud	**0.64** (0.03)	11.74 (1.61)

Note: Interface elevation and layer thickness are average values of five replicates. Standard deviations are given in parentheses.

a The air-water interface (water surface) and the water layer thickness (water depth) only account for the day of measurement 3 April 2012;

b HP (Hydra Probe) Stop is the submersion end due to mechanical resistance in compact mud.

**Table 2.** Mean interface elevation (msl) and mean layer thickness (m) indicated by changes in *PR* to delineate the lake bed surface and shallow lake bed sediment layers in point P053.

	**Interface**			**Layer Thickness**	
	
	**Elevation (msl)**	***PR* (MPa)**		**Thickness (m)**	***PR* (MPa)**
Mud-Lakebed	**113.08** (0.01)	0.70 (0.07)	Mud total ^a^	**0.72** (0.02)	
Bed Layer	**112.78** (0.02)	1.33 (0.21)	Bed Layer	**0.3** (0.02)	−0.60 (0.22)

Note: Interface elevation and layer thickness are average values of at least three replicates. Standard deviations are given in parentheses.

a The total thickness of the mud layer considers the water-mud interface (Table 1) at 113.80 msl.
